# A new species of *Ithome* Chambers (Lepidoptera, Cosmopterigidae, Chrysopeleiinae) from the Atacama Desert revealed by morphology and DNA barcodes

**DOI:** 10.3897/zookeys.912.47562

**Published:** 2020-02-17

**Authors:** Sebastián Espinoza-Donoso, Dante Bobadilla, Wilson Huanca-Mamani, Marcelo Vargas-Ortiz, Héctor A. Vargas

**Affiliations:** 1 Departamento de Recursos Ambientales, Facultad de Ciencias Agronómicas, Universidad de Tarapacá, Casilla 6-D, Arica, Chile; 2 Departamento de Producción Agrícola, Facultad de Ciencias Agronómicas, Universidad de Tarapacá, Casilla 6-D, Arica, Chile; 3 Programa de Doctorado en Sistemática y Biodiversidad, Departamento de Zoología, Facultad de Ciencias Naturales y Oceanográficas, Universidad de Concepción, Concepción, Chile

**Keywords:** Florivorous larvae, *Ithome concolorella* (Chambers, 1875), *Ithome tiaynai* Vargas, 2004, *Prosopis tamarugo* Phil.

## Abstract

Morphology and DNA barcode sequences were used to assess the taxonomic status of a micro-moth of the genus *Ithome* Chambers, 1875 (Lepidoptera, Cosmopterigidae, Chrysopeleiinae), whose larvae feed on inflorescences of *Prosopis tamarugo* Phil. (Fabaceae), a tree native to the Pampa del Tamarugal, Atacama Desert, northern Chile. As a result, *Ithome tamarugensis* Vargas, **sp. nov.** is described and illustrated. Its genitalia are remarkably similar to those of *Ithome tiaynai* Vargas, 2004 from coastal valleys of the Atacama Desert. However, the two species can be recognized by the shape of the phallus in males and the shape of the antrum and ductus bursae in females. The genetic distance between DNA barcodes of *I. tamarugensis* and *I. tiaynai* was 3.0–3.3% (K2P), and a maximum likelihood analysis indicated that they are in reciprocally monophyletic clusters, providing additional support for the heterospecific status suggested by morphology.

## Introduction

The Atacama Desert harbors a distinctive biota characterized by some endemic plants and animals (e.g. Saldivia and Faúndez 2014; Valladares-Faúndez et al. 2018) despite being the most arid desert in the world (Clarke 2006). Among its singular and amazing environments is the Pampa del Tamarugal, a plain at about 1000 m elevation in the Tarapacá Region, northern Chile, whose name is due to the presence of the endemic *Prosopis tamarugo* Phil. (Fabaceae), a tree locally known as Tamarugo. This tree is key in most of the regular ecological processes in the Pampa del Tamarugal, providing food, refuge, and other services to other species (Lopez-Calleja and Estades 1996; Vargas 2007; Carevic et al. 2013). In addition, humans have used this tree in several ways, such as for livestock feed (Zelada 1986). As fruits and leaves are the main organs consumed, serious problems can be triggered by outbreaks of phytophagous insects, among which florivorous lepidopterans are especially important (Vargas and Bobadilla 2000).

*Ithome* Chambers, 1875 (Lepidoptera, Cosmopterigidae, Chrysopeleiinae) is a New World genus of micro-moth with 19 species currently described. Eighteen species of *Ithome* have their type locality on mainland America and one in the Galápagos Islands (Hodges 1961, 1978, 1997; Becker 1984; Landry 2001; Vargas 2004). Although the biology of many species of *Ithome* remains unknown, when the host plants are known, the larvae mainly feed on inflorescences of trees of the family Fabaceae (Hodges 1978).

Two of the species of *Ithome* originally described from the southern United States have expanded their ranges outside of mainland America and are pests of Fabaceae. *Ithome concolorella* (Chambers, 1875), described from Texas, became a pest of *Acacia farnesiana* (L.) Willd. and *Prosopis chilensis* (Mol.) Stuntz in the Hawaiian Islands because feeding by its larvae reduces the flower production of these trees, which are important nectar sources for honey bees (Namba 1956). *Ithome lassula* Hodges, 1962, with its type locality in Florida, is currently established in Australia and Cuba, where its larvae feed on inflorescences of *Leucaena leucocephala* (Lam.) de Wit (Common and Beattie 1982; Alonso et al. 2015).

*Ithome tiaynai* Vargas, 2004 is the only representative of the genus currently described from Chile. Its larvae feed on inflorescences of *Acacia macracantha* Willd. (Fabaceae) in coastal valleys of the Atacama Desert (Vargas 2004), reaching higher densities on trees growing in well-preserved habitats than in highly human-modified ones (Vargas and Parra 2009). In addition, an unidentified species of *Ithome* was mentioned by Hodges (1978) from central Chile, and another, pest species, is known from the Pampa del Tamarugal, where its larvae feed on inflorescences of *P. tamarugo*, affecting the fruit production (Vargas et al. 1986). This last species was referred to as *I. concolorella* in the agricultural literature (Artigas 1994). However, because *I. concolorella* is a Northern Hemisphere species (Hodges 1978), the identity of the *Ithome* from the Pampa del Tamarugal has remained doubtful.

We used morphology and DNA barcode sequences (sensu Hebert et al. 2003) to assess the taxonomic status of the *Ithome* from the Pampa del Tamarugal. The two character sources revealed that this micro-moth represents a new species whose formal description is provided here.

## Materials and methods

### Sampling and rearing

Larvae of *Ithome* were collected on inflorescences of *P. tamarugo* in La Tirana village, at about 1000 m elevation in Tamarugal Province, Tarapacá Region, northern Chile, in August 2018 and September 2019. The inflorescences with larvae were placed in plastic vials with paper towel at the bottom and brought to the laboratory, where additional inflorescences were provided until the larvae finished feeding and pupated. The vials were observed daily until adult emergence. The adults were mounted and their abdomens were removed and placed in hot KOH 10% for a few minutes for dissection of their genitalia, which were stained with Chlorazol black and Eosin Y and slide mounted with Euparal. Description of genitalia follows Hodges (1978). Pupae of *I. tiaynai* were reared from larvae collected in March 2016 on inflorescences of *A. macracantha* in the type locality of this micro-moth. Three pupae of the each of the two *Ithome* species were placed in ethanol 95% at −20 °C until DNA extraction.

### DNA extraction, sequencing and analysis

Genomic DNA was extracted following the procedures described by Huanca-Mamani et al. (2015) from pupae of the *Ithome* pest of *P. tamarugo* and *I. tiaynai*. Genomic DNA was sent to Macrogen Inc., South Korea, for purification, PCR amplification, and sequencing of the DNA barcode fragment with the primers LCO-1490 and HCO-2198 (Folmer et al. 1994) using the PCR program described in Escobar-Suárez et al. (2017). The software MEGA7 (Kumar et al. 2016) was used to perform the sequence alignment by the ClustalW method, to estimate the sequence divergence by the Kimura 2-Parameter (K2P) method, and to calculate the phylogenetic tree using the maximum likelihood (ML) approach with 1000 bootstrap replications. The nucleotide substitution model was chosen using the lowest Bayesian information criterion (BIC) value. The sequence used as outgroup belongs to *Ithome curvipunctella* (Walsingham, 1892), the only congeneric with barcode sequences available in BOLD (Ratnasingham and Hebert 2007). To detect the presence of phylogenetic signal, substitution saturation analysis was previously performed using the Xia test (Xia et al. 2003) in the Dambe 7.2.1 program (Xia 2018).

### Abbreviations of institutional collections

**MNNC** Museo Nacional de Historia Natural de Santiago, Santiago, Chile

**IDEA** Colección Entomológica de la Universidad de Tarapacá, Arica, Chile

## Results

### Molecular analysis

**Figure 1.**
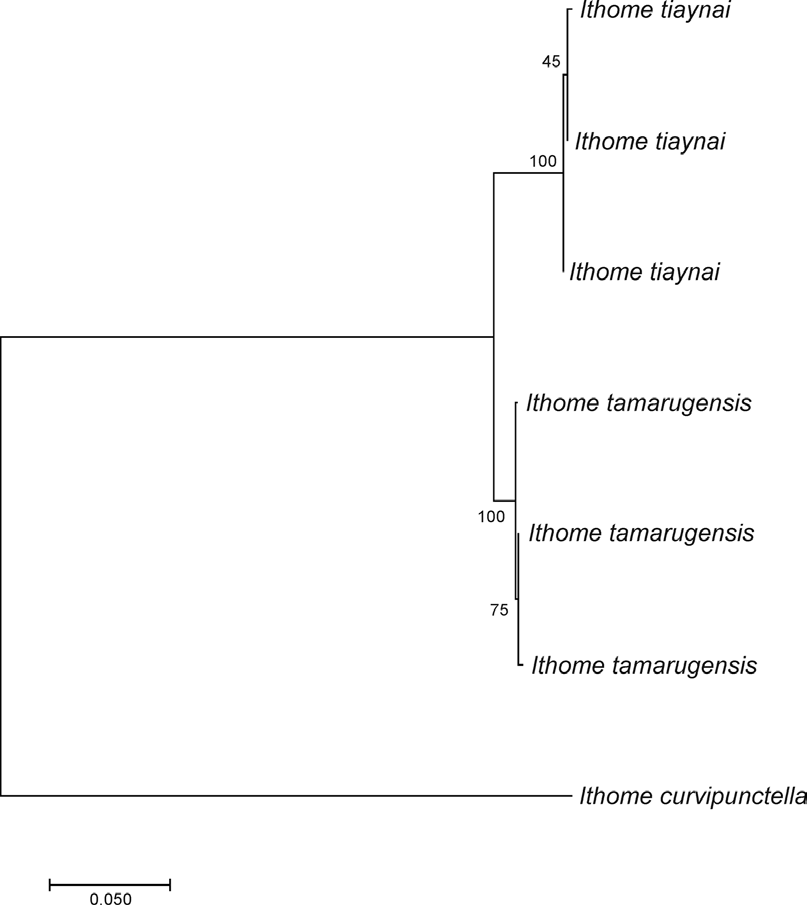
Maximum likelihood tree of the haplotypes of the DNA barcode fragment of *Ithome tamarugensis* Vargas, sp. nov. and *Ithome tiaynai*. Numbers indicate bootstrap percentages (1000 replicates). One sequence of *Ithome curvipunctella* was used as outgroup.

Six DNA barcode sequences were obtained (Table 1), three of *Ithome* from the Pampa del Tamarugal and three of *I. tiaynai*, each representing a different haplotype. Intra- and interspecific genetic divergence was 0.2–0.3 and 3.0–3.3% (K2P), respectively. The sequence alignment length was 657 bp. Codon stops were not detected and substitution saturation was not found, indicating that the data set was suitable for phylogenetic analysis. The substitution model used was TN92 + G (with the lowest BIC value). A maximum likelihood analysis separated the haplotypes of the two species into reciprocally monophyletic clusters (Fig. 1).

**Table 1.** Sequences used in the analyses.

Species	BOLD accession	GenBank accession	Country
*Ithome curvipunctella* (Walsingham, 1892)	BBLOB1350-11		USA
*Ithome tamarugensis* Vargas, sp. nov.		MN586873	Chile
*I. tamarugensis*		MN586874	Chile
*I. tamarugensis*		MN586875	Chile
*Ithome tiaynai* Vargas, 2004		MN586876	Chile
*I. tiaynai*		MN586877	Chile
*I. tiaynai*		MN586878	Chile

### Taxonomy

#### Ithome tamarugensis

Taxon classificationAnimaliaLepidopteraCosmopterigidae

Vargas sp. nov.

332882FC-57BA-5093-AEE6-F491D9109FE3

http://zoobank.org/43BEAA78-9AA8-43BB-BD05-6BC197292B7F

Figures 2 , 3 , 4

Ithome sp. (Vargas et al. 1986; Vargas and Bobadilla 2000)
Ithome concolorella ; misidentification (Artigas 1994)

##### Type locality.

Chile, Tarapacá Region, Tamarugal Province, La Tirana village, 20°20'S, 69°39'W.

##### Type material.

Holotype male, pinned, genitalia slide HAV-1307. Original labels: “Chile, Tamarugal, La Tirana, emerged October, 2019, H.A. Vargas coll.”, “ex-larva inflorescence *Prosopis tamarugo*, collected September, 2019”, “Holotype / *Ithome* / *tamarugensis* / Vargas” [red handwritten label] (MNNC).

##### Other material.

Paratypes (Five males, five females). One male (genitalia slide HAV-1309), two females (genitalia slides HAV-1308, 1310), same data as for holotype (MNNC). Four males (genitalia slides HAV-1311, 1313, 1315, 1317), three females (genitalia slides HAV-1312, 1314, 1316), same data as for holotype (IDEA).

##### Diagnosis.

The mainly shiny black adults of *I. tamarugensis* resemble those of *I. tiaynai*, the only other Chilean congeneric. In addition, the genitalia of the two species are outstandingly similar. However, the male of *I. tamarugensis* has the phallus dorsally excavated in the middle, slightly broadened subapically, and with a deep cleft apically. In contrast, the phallus of *I. tiaynai* is parallel sided in the middle, slightly straightening subapically, and with a small cleft apically. The female of *I. tamarugensis* has a short cylindrical antrum and coiled ductus bursae. In contrast, the female of *I. tiaynai* has a mainly undifferentiated antrum and conic ductus bursae. Although signa were not mentioned in the original description of *I. tiaynai*, recent observations have revealed the presence of two signa similar to those found in *I. tamarugensis*.

##### Description.

**Figure 2.**
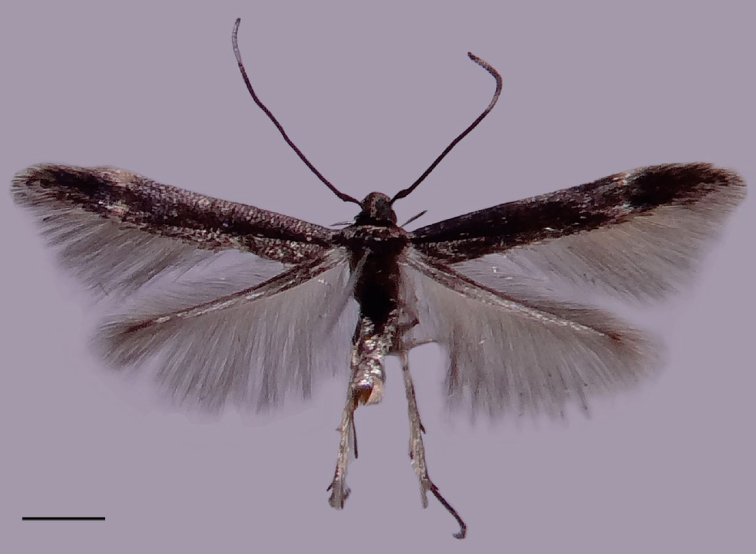
Holotype of *Ithome tamarugensis* Vargas sp. nov. in dorsal view. Scale bar: 1 mm.

**Male** (Fig. 2). Forewing length 4.4–4.6 mm.

Head. Shiny dark grey on face, shiny black dorsally. Haustellum shiny dark grey. Labial palpus shiny black. Antenna shiny black, filiform, about 3/4 wing length; pecten formed by a dark brown scale placed near base of scape.

Thorax. Shiny black dorsally, brownish grey laterally. Foreleg mainly shiny black; coxa brownish grey laterally; brownish grey at middle and apex of tibia and apex of tarsomeres. Midleg mainly shiny black; two brownish grey tibial spurs; brownish grey at apex of tarsomeres. Hindleg mainly shiny black laterally, brownish grey medially; tibia with longitudinal stripe of short, hair-like, yellowish-grey scales dorsally; four brownish-grey tibial spurs; tarsomeres mainly brownish grey with a few darker areas. Forewing shiny black with slightly differentiated (or sometimes absent) brownish-grey transverse stripe subapically; fringe brownish grey. Hindwing shiny black on basal half, brownish grey on distal half; fringe brownish grey.

**Figure 3.**
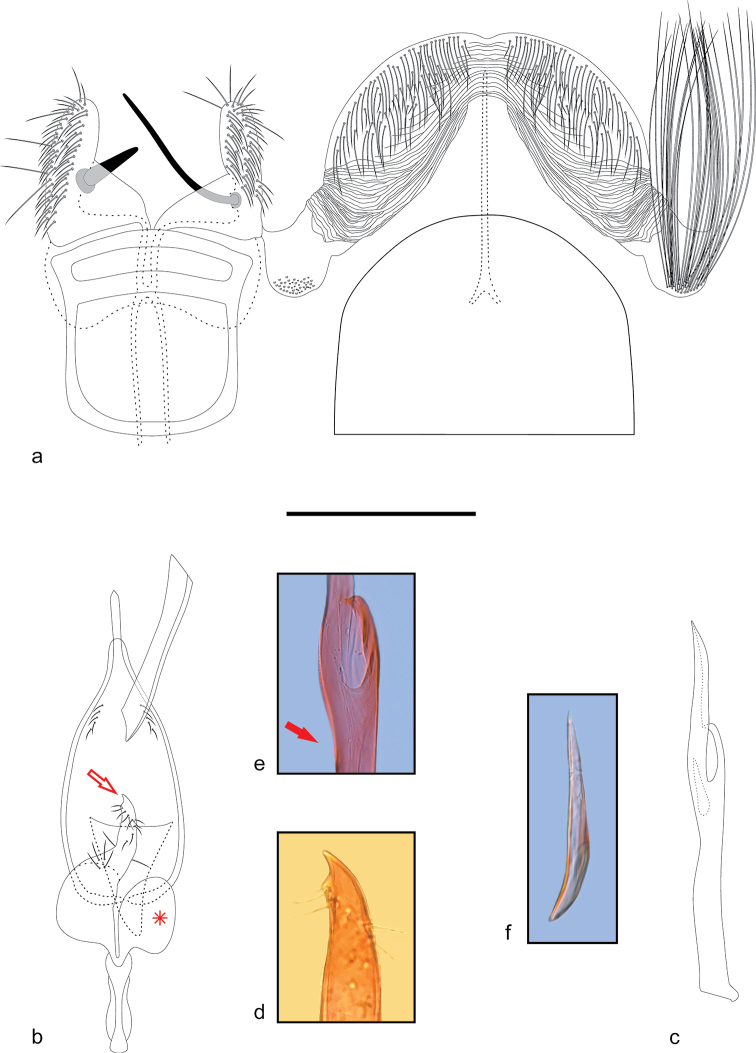
Male genitalia and pregenital abdominal segments of *Ithome tamarugensis* Vargas, sp. nov. **A** Abdominal segments VII and VIII **B** genitalia in ventral view, phallus removed, asterisk shows the right valva **C** phallus in lateral view **D** apex of the hook of the left valva (marked with arrow in **B**) **E** apex of phallus, arrow shows the dorsal excavation **F** cornutus. Scale bar: 0.1 mm.

Abdomen (Fig. 3a). Mainly brownish grey; shiny black on terga I–IV; lateral membrane of segment VII with a pocket of yellowish-brown, hair-like scales exceeding slightly the posterior margin of tergum VIII. Tergum VII mainly not modified, posterior margin broadly convex. Sternum VII modified, well-sclerotized margins, a transverse stripe close to the posterior margin divides the sternum into a square-shaped anterior part and a posterior transverse band; anterolateral margin slightly convex; posterior part of lateral margin laterally projected, posterior margin slightly convex. Tergum VIII hood-shaped; anterior margin broadly concave, posterior margin broadly convex, slightly excavated in the middle; narrow, sclerotized rod medially, anterior end bifurcated. Sternum VIII strongly modified, partially overlapped with sternum VII; longitudinally divided by a sharp posterior excavation that reaches the anterior margin; two narrow sclerotized rods medially in the middle of anterior margin, length similar to rod of tergum VIII; two finger-like posterior projections of similar length, with right one slightly narrower; both projections with an inward spine of differing length; the left projection with a short, robust, and tapering spine medially near middle, the right projection with a long, narrow, and straight spine medially, bent near base.

***Male genitalia*** (Fig. 3b–f). Uncus short, slightly shorter than the left spine on sternum VII, narrow, parallel sided, apex suddenly pointed. Tegumen narrow, somewhat forceps-like, slightly broadened ventrally. Saccus about 1.5 times length of uncus, with a longitudinal ventral carina and a pair of convex lateral projections. Subscaphium as a narrow stripe, slightly sclerotized, and with a few small setae on the membrane near base of subscaphium. Valvae asymmetrical; left valva lobular (Fig. 3b, arrow), a slightly curved pointed hook distally (Fig. 3d), a few short setae near apex of hook; right valva lobular without hook. Phallus (Fig. 3c) cylindrical, dorsally excavated at middle (Fig. 3e, arrow), slightly broadened subapically; apex with a deep cleft and a knife-like projection. Vesica with a conic cornutus (Fig. 3f), similar in size to apical cleft of phallus.

**Female.** Similar to male in size and coloration. Hindwing brownish grey; fenulum with three acanthae. Abdomen brownish grey. Separation between VI and VII segments slightly differentiated.

**Figure 4.**
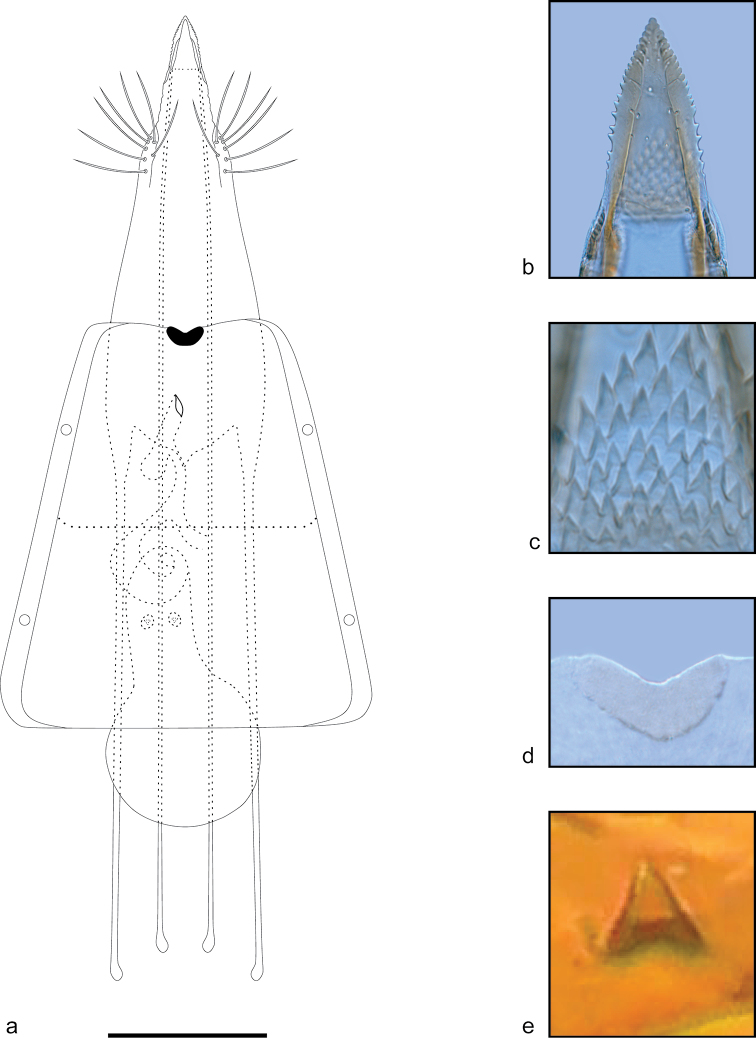
Female genitalia of *Ithome tamarugensis* Vargas, sp. nov. **A** Female genitalia in ventral view **B** papillae analis **C** ornamentation of intersegmental membrane between papillae and segment VIII **D** lamella postvaginalis **E** signum. Scale bar: 0.1 mm.

***Female genitalia*** (Fig. 4a–e). Papillae analis well sclerotized, fused medially, forming a triangular plate with serrated lateral margin (Fig. 4b). Intersegmental membrane between papillae and segment VIII with triangular spinules (Fig. 4c). Posterior apophysis rod-shaped, narrow, elongated, about three times length of tergum VIII. Anterior apophysis rod-shaped, about 2/3 length of posterior apophysis. Tergum VIII slightly more sclerotized and with a few long setae posterolaterally. Lamella postvaginalis as a small semicircular plate in middle of posterior margin of sternum VII (Fig. 4d). Ostium bursae as a narrow longitudinal slit on middle of sternum VII. Antrum cylindrical, apex rounded. Ductus bursae arising subapically on antrum, membranous, coiled. Corpus bursae pear-shaped, membranous, with two small conical signa close to the base (Fig. 4e).

##### Geographic distribution.

**Figure 5.**
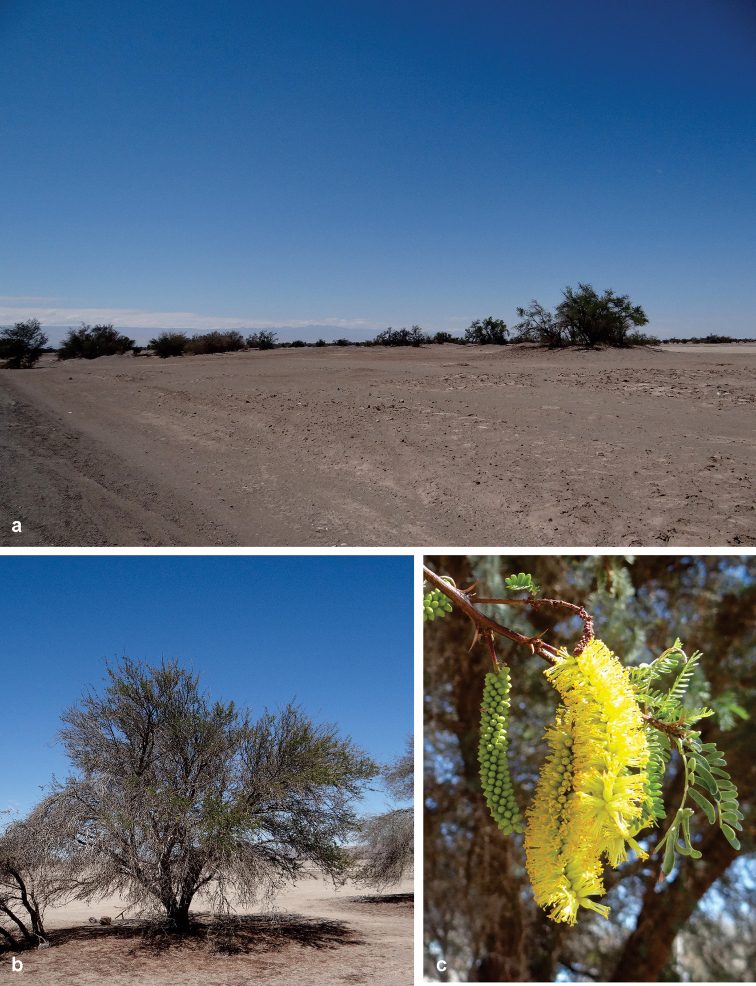
Habitat and host plant of *Ithome tamarugensis* Vargas, sp. nov. in the Atacama Desert. **A** Habitat near La Tirana village, Pampa del Tamarugal, northern Chile **B** a tree of tamarugo, *Prosopis tamarugo* (Fabaceae) **C** inflorescences of *P. tamarugo* at bud and flowering stages.

*Ithome tamarugensis* is known from the Pampa del Tamarugal (Fig. 5a), Atacama Desert of northern Chile.

##### Host plants.

All the specimens of *I. tamarugensis* examined in this study were reared from larvae collected on *P. tamarugo* (Fig. 5b, c). This tree is the main host plant of *I. tamarugensis*, although its larvae also feed on inflorescences of at least two other species of *Prosopis*, P. alba Griseb. var. alba and P. strombulifera (Lam.) Benth. var. strombulifera (Vargas and Bobadilla 2000).

##### Etymology.

The specific epithet is derived from the Pampa del Tamarugal, where the type locality of *I. tamarugensis*, La Tirana village, is located.

## Discussion

Despite the extreme aridity of the Atacama Desert, some reports in the last decades suggest that it harbors a distinctive micro-moth fauna associated with native plants (e.g. Clarke 1987; Pereira et al. 2017; Vargas-Ortiz et al. 2019), which is reinforced by this study in which the taxonomic status of the *Ithome* pest of *P. tamarugo* in the Pampa del Tamarugal was assessed using morphology and DNA barcodes. The two character sources revealed that this micro-moth represents a previously undescribed species, here named *I. tamarugensis*. This discovery allows us to conclude that the previous record of *I. concolorella* in Chile (Artigas 1994) was based on a misidentification of *I. tamarugensis*. These two species can be clearly separated by the morphology of the asymmetrical posterior projections of the male sternum VIII. In *I. concolorella*, the left projection has an apical spine and the right projection is unarmed (Hodges 1961, 1978). In *I. tamarugensis*, each of the two projections has a medial spine, which is short in the left projection and long in the right one (Fig. 3a).

Although there are no previous studies dealing with phylogenetic relationships of the species of *Ithome*, the morphology suggests that *I. tamarugensis* and *I. tiaynai* are closely related. First, the highly specialized sternum VIII of the male abdomen is indistinguishable among them, but clearly different from this structure in the remaining species of the genus (Hodges 1961, 1978; Landry 2001). Second, the remarkably similar genitalia of *I. tamarugensis* and *I. tiaynai* are distinguishable only after a careful examination of very fine morphological traits. In addition, although the ML analysis included sequences of only three species of *Ithome*, the clusters in which the sequences of *I. tamarugensis* and *I. tiaynai* were reciprocally grouped were highly supported by bootstrap (Fig. 1), and the genetic distance between the two species using the COI marker is comparable to those reported for other morphologically close species of Lepidoptera (e.g. Hausmann et al. 2009; Matson and Wagner 2017; Ullah et al. 2017; Müller 2018; Pfeiler et al. 2018). Accordingly, although very preliminarily, the genetic analysis also provides evidence in support of a close evolutionary relationship between the two Chilean species of *Ithome*.

The current data suggest that the geographic ranges of the two species of *Ithome* of northern Chile are restricted to different ecological zones of the Atacama Desert, the coastal valleys in the case of *I. tiaynai*, and the Pampa del Tamarugal in the case of *I. tamarugensis*. A similar geographic pattern has been described for two morphologically close micro-moths of the genus *Cryptophlebia* Walsingham (Tortricidae), with *C. cortesi* Clarke, 1987 restricted to the coastal valleys and *C. saileri* Clarke, 1987 to the Pampa del Tamarugal (Clarke 1987). This coincident pattern in two distantly related families suggests that allopatry could have been an important mechanism underlying diversification in some groups of micro moths in these arid environments. Genetic differentiation between isolated populations has been shown in at least two other micro-moths of the Atacama Desert (Escobar-Suárez et al. 2017; Vargas-Ortiz et al. 2018), which agrees with a scenario of allopatric speciation. However, further comprehensive phylogenetic studies, including a detailed taxon sampling and analyses of morphological characters and biparental (or male-linked) molecular markers, will be needed to reconstruct the diversification process in the genus *Ithome* and to assess the close evolutionary relationship between *I. tamarugensis* and *I. tiaynai* that is here suggested.

## Supplementary Material

XML Treatment for Ithome tamarugensis
